# Triterpenoids from *Euphorbia maculata* and Their Anti-Inflammatory Effects

**DOI:** 10.3390/molecules23092112

**Published:** 2018-08-22

**Authors:** Yi Sun, Liang-liang Gao, Meng-yue Tang, Bao-min Feng, Yue-hu Pei, Ken Yasukawa

**Affiliations:** 1 Institute of Chinese Materia Medica, China Academy of Chinese Medical Sciences, Beijing 100700, China; m18656251050@163.com; 2 School of Pharmacy, Nihon University, 7-7-1, Narashinodai, Funabashi, Chiba 274-8555, Japan; 3 College of Food and Medicine, Anhui Science and Technology University, Fengyang 233100, China; Gll8179@163.com; 4 Pharmacy College, Harbing Medical University, Harbin 150081, China; 5 School of Life and Sciences and Biotechnology, Dalian University, Dalian 116622, China; fengbaomin@dlu.edu.com

**Keywords:** *Euphorbia maculata*, natural products, triterpenoids, anti-inflammatory activity

## Abstract

*Euphorbia maculata* is a medicinal plant of the Euphorbiaceae family, which can produce anti-inflammatory and cancer chemopreventive agents of triterpenoids. The present study reports on the bioactive triterpenoids of this plant. Two new lanostane-type triterpenoids, named (3*S*,4*S*,7*S*,9*R*)-4-methyl-3,7-dihydroxy-7(8→9) *abeo*-lanost-24(28)-en-8-one (**1**) and 24-hydroperoxylanost-7,25-dien-3β-ol (**2**), together with 15 known triterpene derivatives, were isolated from *Euphorbia maculata*. The structures of the new compounds were determined on the basis of extensive spectroscopic data (UV, MS, ^1^H and ^13^C-NMR, and 2D NMR) analysis. All tetracyclic triterpenoids (**1**–**11**) were evaluated for their anti-inflammatory effects in the test of TPA-induced inflammation (1 μg/ear) in mice. The triterpenes exhibited significant anti-inflammatory activities.

## 1. Introduction

*Euphorbia maculata* L. is a plant of the Euphorbiaceae family, which is widely used as folk medicine throughout the world, especially in China, Japan, and Korea [1]. Many species of the genus *Euphorbia* have a variety of applications in traditional Chinese medicines (TCM). *E. maculata* is an annual herb that is commonly used for the treatment of diarrhea, hemoptysis, hematuria, and sore swollen [2]. Extracts of this plant has an antiplatelet activity via suppressing thromboxane B2 formation [3]. Previous phytochemical investigations of *E. maculata* yielded tannins, flavonol glycoside, and triterpenoids [4,5,6,7].

Naturally occurring triterpenoids possesses anti-tumor, anti-inflammatory, anti-proliferative, and anti-HIV activities. Many triterpenoids of different skeletons exhibited anti-inflammatory and inhibitory activities on tumor promotion induced by TPA [8,9,10]. As many tumor promoters can cause inflammatory symptoms, the inhibitory effect against TPA-induced ear inflammation has a close correlation with the inhibition of TPA-induced tumor promotion in a two-stage carcinogenesis. Thus, we chose the medicinal plants of the genus *Euphorbia* (Euphorbiaceae) which contained triterpenoids to study their chemical constituents and pharmacological activity [11]. We found that the lanostane-type triterpenes of *E. kansui* markedly possessed the inhibitory activity against TPA-induced inflammation, and the predominant triterpene in this plant, euphol, significantly inhibited the tumor-promoting activity induced by TPA [12].

In the course of our continuing search for anti-inflammatory compounds from plants [13,14,15,16], we focused on the *Euphorbia maculata* due to their medicinal use and began to clarify their anti-inflammatory principles. Herein, we describe the structural elucidation of the new triterpenoids and the anti-inflammatory activities of all tetracyclic isolated triterpenoids.

## 2. Results and Discussion

### 2.1. Triterpenoids of Euphorbia maculata

**Figure 1:**
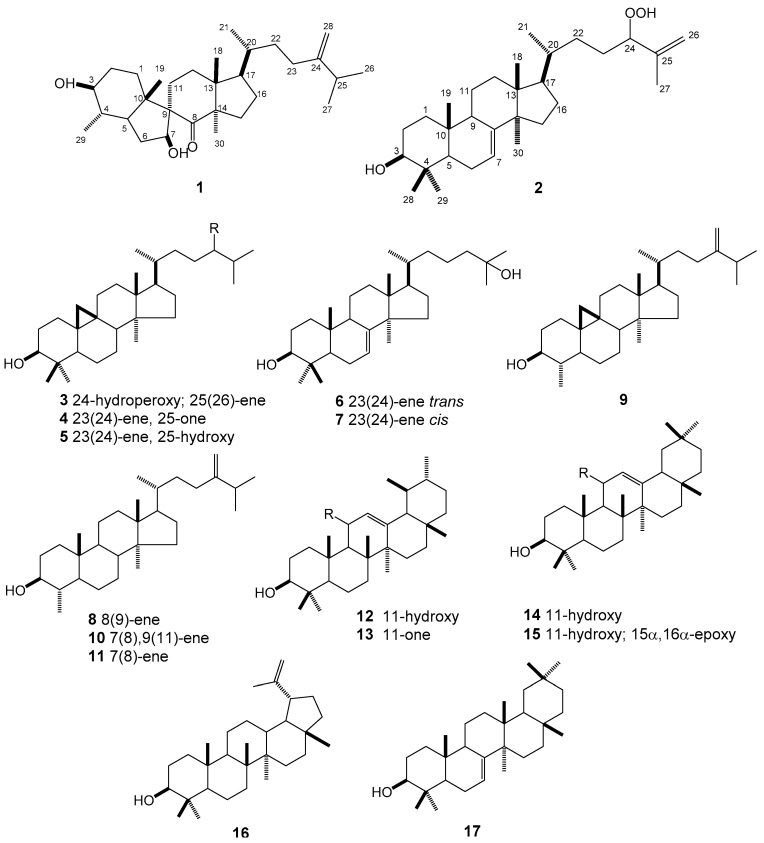
The chemical structures of compounds **1**–**17** isolated from *Euphorbia maculata.*

We investigated the *n*-hexane extract of *E. maculata* by the bioassay-guided fractionation method. Seventeen triterpene derivatives (**1**–**17**) (Figure 1), including a new spiro-triterpenoid (**1**) and a new lanostane triterpenoid (**2**), were isolated. Compounds **3**–**17** were identified to be 3-hydroxycycloart-25-ene-24-hydroperoxide (**3**) [17], 3β-hydroxy-26-nor-9,19-cyclolanost-23-en-25-one (**4**) [18], cycloart-23en-3β,25-diol (**5**) [19], (23*E*)-3β,25-dihydroxytirucalla-7,23-diene (**6**) [20], (23*Z*)-3β,25-dihydroxy-tirucalla-7,23-diene (**7**) [21], Obtusifoliol (**8**) [22], cycloeucalenol (**9**) [23], 4α,l4α-dimethyl-5α-ergosta-7,9(11),24(28)-trien-3β-ol (**10**) [24], gramisterol (**11**) [25], urs-12-ene-3β,11α-diol (**12**) [26], Neoilexonol (**13**) [27], 12-Oleanene-3β,11β-diol (**14**) [28], (3β,15α,16α)-15,16-epoxy-Olean-12-en-3-ol (**15**) [29], lupeol (**16**) [30], multiflorenol (**17**) [31] by compared spectroscopic data and physicochemical properties with those reported in the literatures. The new compounds were determined by means of mass spectrometry and extensive 1D and 2D NMR.

### 2.2. Structures

**Figure 2:**
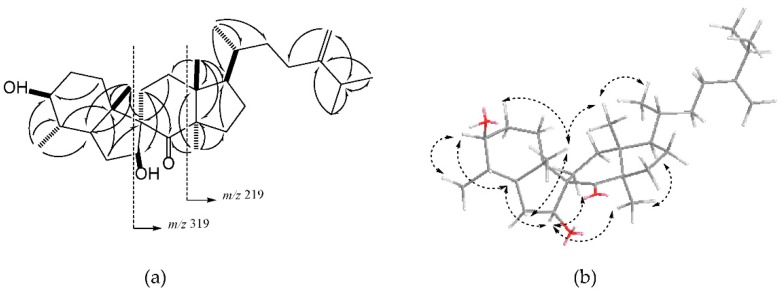
(**a**) Key HMBC correlations and EIMS of **1**; (**b**) Key NOESY correlations of **1**.

Compound **1** was isolated as a white solid. Its HREIMS spectrum showed a molecular ion peak at *m*/*z* 458.3759 [M^+^], corresponding to the molecular formula C_30_H_50_O_3_. The ^1^H-NMR (Table 2, Figure S1) displayed characteristic signals for seven methyl groups [*δ*_H_ 0.66 (3H, s), 0.93 (3H, d, *J* = 6.5 Hz), 0.95 (3H, d, *J* = 6.2 Hz), 1.02 (3H, d, *J* = 6.7 Hz), 1.03 (3H, d, *J* = 6.8 Hz), 1.21 (3H, s), 1.48 (3H, s)], two oxygenated methine protons [*δ*_H_ 3.05 (1H, td, *J* = 10.0, 4.8 Hz) and 4.35 (1H, m)], and two olefinic protons [*δ*_H_ 4.66 (1H, d, *J* =1.2 Hz) and 4.72 (1H, d, *J* = 0.9 Hz)]. The ^13^C-NMR (Table 2, Figure S2) and HMQC spectra (Figure S3) disclosed the existence of thirty carbons including a ketone (*δ*_C_ 215.1), two olefins (*δ*_C_ 156.5 and 106.2), and two oxygenated methines (*δ*_C_ 80.5 and 77.5). The aforementioned data suggested that **1** was a triterpenoid (Figure 2). In the ^1^H-^1^H COSY spectrum (Figure S5), five fragments were obtained according to the spin systems of H_2_-1/H_2_-2/H-3/H-4/H-5/H_2_-6/H-7, H_2_-11/H_2_-12, H_2_-15/H_2_-16/H-17, H_3_-21/H-20/H_2_-22/H_2_-23, and H-25/ H_3_-26/ H_3_-27. The HMBC correlations (Figure S4) from H-4 and H_3_-29 to C-3, and from H_2_-6 and H_2_-11 to C-7 indicated that the two hydroxyl groups were located at C-3 and C-7, respectively. Furthermore, the HMBC correlations from both H_3_-19 and H_2_-6 to C-5, C-9, C-10, and from H-7 to C-5, C-9 and C-10, as well as the ^1^H-^1^H COSY cross-peaks of H_2_-6/H-7 and H_2_-11/H_2_-12 suggested that the quaternary carbon C-9 was the connectivity of a spiro center. The ketone group was substituted at C-8, which was indicated by the HMBC correlations from H_3_-30 to C-8, C-13, and C-14, and from H_2_-11 to C-8. The EIMS of **1** displayed two characteristic peaks of spiro-triterpenoid at 319.2 [C_21_H_35_O_2_] and 219.2 [C_16_H_27_], together with *m*/*z* 440 [M − H_2_O]^+^ and 422 [M − 2H_2_O]^+^. The relative configuration of **1** was determined by NOESY spectrum (Figure 2, Figure S6). The NOESY correlations between H_3_-19 and H-2β, H-6β, and H_3_-18 indicated that these protons were at the same orientation. Furthermore, the orientations of the two hydroxyl groups were deduced as β on the basis of the NOE correlations between H-7 and H-5α, H-11α, and H_3_-30, and between H-3 and H-5α, and H_3_-29. The above NOE correlations also suggested that C-9 was *R* configuration. The C-ring was supposed to be a boat configuration based on the NOE correlations of H-5α with H-11β and H-17α with H_3_-30, which was the same as that of spiroinonotsuoxodiol. Based on the comparison of its NMR data with that of a spiro-triterpene in the literature [32], the structure of **1** was determined to be (3*S*,4*S*,7*S*,9*R*)-4-methyl-3,7-dihydroxy-7(8→9) *abeo*-lanost-24(28)-en-8-one (Figure 1).

Compound **2** was obtained as a white solid. The molecular formula of **2** was determined to be C_30_H_50_O_3_ ([M]^+^ *m*/*z* 458.3755; Calcd. 458.3760) on the basis of HREIMS and NMR data. The ^1^H-NMR spectrum (Table 2, Figure S7) exhibited six methyl singlets at *δ*_H_ 0.75 (3H, s), 0.81 (3H, s), 0.86 (3H, s), 0.98 (6H, s), 1.73 (3H, s), a methyl group doublets at *δ*_H_ 0.75 (3H, d, *J* = 7.4 Hz), two oxygenated methine protons at *δ*_H_ 3.24 (1H, dd, *J* = 11.7, 4.6 Hz) and 4.21 (1H, t, *J* = 8.0 Hz), and two olefinic protons at *δ*_H_ 5.02 (1H, m) and 5.26 (1H, dd, *J* = 7.6, 3.3 Hz). Analysis the ^13^C-NMR (Table 2, Figure S8) and HMQC spectra (Figure S9) displayed signals corresponding to six quaternary carbons, including two olefinic (*δ*_C_ 145.7, 143.8) and four aliphatic carbons (*δ*_C_ 34.9, 38.9, 43.5, 51.1), ten methylene groups with corresponding carbons between *δ*_C_ 18.1 and *δ*_C_ 37.2, a further olefinic group at *δ*_C_ 114.2, seven methine groups at *δ*_C_ 36.0–117.8, and seven methyl groups at *δ*_C_ 13.1–27.7. These NMR data suggested that **2** was a lanostane-type triterpenoid [33]. The HMBC correlations (Figure S10) from H_2_-1, H-5 and H_3_-28 to C-3 indicated that an -OH group was present at C-3 (*δ*_C_ 79.7) (Figure 2). The HMBC correlations from H-5 to C-7, and from H_2_-11 to C-8 confirmed a double bond at C-7 (*δ*_C_ 117.8) and C-8 (*δ*_C_ 145.7). HMBC correlations from H_2_-23, H_2_-26 and H-27 to C-24 and chemical shifts of C-24 (*δ*_C_ 90.1 and *δ*_H_ 4.21) suggested that a hydroperoxy group was located at C-24. The NOESY spectrum of **2** exhibited correlations between H-3α and H-5α, and between H-5α and H-6α, supporting the β-orientation of the 3-OH group (Figure 2, Figure S12). The NOESY correlations between H_3_-30 and H-17α, and between H-17α and H_3_-21 indicated that H-17 was α-oriented and C-20 was *R* configuration. Therefore, compound **2** was determined to be 24-hydroperoxylanost-7, 25-dien-3β-ol, whose structure is similar to **3** (Figure 1).

### 2.3. Anti-Inflammatory

The *n*-hexane extract of the whole plant of *E. maculata* inhibited the inflammatory ear oedema induced by TPA with the ID_50_ (50% inhibitory dose) value of 0.8 mM. All the tetracyclic triterpenoids were evaluated with respect to their anti-inflammatory activity against TPA-induced inflammation in mice, and the inhibitory effects were compared with indomethacin, a commercially available anti-inflammatory drug, as shown in Table 1. Most of the tetracyclic triterpenoids exhibited a potent inhibitory activity, with ID_50_ values in the range of 87.1–1087 nM/ear, of which compound **8** showed the most potent inhibitory activity higher than indomethacin (ID_50_ 838 nM/ear). The results indicated that compounds **8**, **10**, and **11** with only one methyl substitution at C-4 and a double bond at C-24 in the side chain possessed a stronger anti-inflammatory activity than the other tetracyclic triterpenoids.

Another tetracyclic triterpene, euphol isolated from *E. kansui* possessed both anti-inflammatory and anti-tumor promoting activities. Various triterpenoids of different skeletons have been demonstrated to inhibit the tumor-promoting activities induced by TPA. The inhibitory activity of euphol was similar to lupeol (**16**) [34]. Thus, we concluded that most triterpenes isolated from *E. maculata* probably inhibited the tumor-promoting activity by TPA.

## 3. Materials and Methods

### 3.1. General Experimental Procedures

Optical rotations were measured on a Jasco DIP-360 polarimeter. UV spectrum was obtained on a Jasco V-550 spectraphotometer (Tokyo, Japan). FAB-MS and HR-FAB-MS were obtained using a JEOLJMS-GC mate spectrometer (JEOL, Tokyo, Japan). NMR spectra were recorded using a JEOL ECA 600 spectrometer (JEOL, Tokyo, Japan), with TMS as an internal standard. Silica gel (230–400 mesh, Merck, Darmstadt, Germany) and Silica gel plates (GF_254_, Merck, Darmstadt, Germany) were used for CC and TLC, respectively. Sephadex LH-20 was purchased from Pharmacia (Uppsala, Sweden). ODS-EP column (250 × 10 mm, Inertsil; GL sciences Inc., Tokyo, Japan) was used for preparative HPLC.

### 3.2. Plant Material

The dried whole plant of *Euphorbia maculata* was collected in Medicinal Plant Garden of College of Pharmacy, Nihon University. The plant was authenticated by Prof. Yasukawa, College of Pharmacy, Nihon University, Chiba, Japan. A voucher specimen has been deposited in the College of Pharmacy, Nihon University, Chiba, Japan.

### 3.3. Extraction and Isolation

The dried whole plant of *E. maculata* (1.5 kg) was ground and extracted with MeOH (3 L × 24 h × 5) at room temperature. After the combined extract was evaporated under reduced pressure at 40 °C, the residue (32.6 g) was suspended in H_2_O and then partitioned with EtOAc. The EtOAc-soluble extract (14.0 g) was partitioned with *n*-hexane–MeOH–H_2_O (19:19:2), giving *n*-hexane- (13.0 g) and MeOH–H_2_O (0.9 g) soluble fractions. The H_2_O fraction was partitioned between *n*-BuOH–H_2_O (1:1), yielding *n*-BuOH (5.9 g) and H_2_O (8.3 g) fractions.

The *n*-hexane extract was subjected to a silica gel column, eluting with a gradient of *n*-hexane/EtOAc, and then monitored on TLC to obtain five major fractions. Fraction 3 (4.8 g) was chromatographed over a silica gel column (eluting with PE/acetone), resulting in six fractions (fr. 4.1–4.6). Fraction 4.2 was further purified by Sephadex LH-20 and reversed-phase HPLC (75–85% ACN/H_2_O) to afford **1** (3 mg). Fraction 4.3 was separated by reversed-phase HPLC (70–90% ACN/H_2_O), yielding **2** (2 mg), **3** (3 mg), **4** (5 mg), **5** (1 mg), **6** (4 mg), **7** (4 mg), **13** (7 mg), and **14** (12 mg). Fraction 4.4 was purified by reversed-phase HPLC (70–85% ACN/H_2_O) to give **8** (2 mg), **9** (2 mg), **12** (5 mg), **15** (4 mg), and **16** (3 mg). Fraction 4 (3.6 g) was isolated by HPLC (70–85%ACN/H_2_O) to yield **10** (5 mg) and **11** (7 mg).

#### 3.3.1. Compound **1**

White solid. [a]${}_{D}^{20}$: −35.5 (*c* 0.1, CHCl_3_). HREIMS *m*/*z*: 458.3759 [M]^+^ (C_30_H_50_O_3_, calcd for 458.3760). EIMS *m*/*z*: 458 [M]^+^, 440 [M − H_2_O]^+^, 425 [M − H_2_O − Me]^+^. ^1^H and ^13^C-NMR spectroscopic data, see Table 2. All NMR spectra of **1** have been provided in the Supplementary Material.

#### 3.3.2. Compound **2**

White solid. [a]${}_{D}^{20}$: +4.2 (*c* 0.1, CHCl_3_). HREIMS *m*/*z*: 458.3755 [M]^+^ (C_30_H_50_O_3_, calcd for 458.3760). EIMS *m*/*z*: 458 [M]^+^, 443 [M − Me]^+^, 425 [M − Me − H_2_O]^+^, 407 [M − Me − 2H_2_O]^+^. ^1^H and ^13^C-NMR spectroscopic data, see Table 2. All NMR spectra of **2** have been provided in the Supplementary Material.

### 3.4. Biological Assay

#### 3.4.1. Animals

Experiments were performed in accordance with the guidelines of the Institutional Animal Care and Use Committee of the College of Pharmacy, Nihon University, Chiba, Japan. Female ICR mice were purchased from Japan SLC Inc., Shizuoka, Japan, and housed in an air-conditioned specific pathogen free room (22–23 °C) lit from 08:00 to 22:00. Food and water were available *ad libitum*.

#### 3.4.2. Assay of TPA Inflammation Ear Edema in Mice

TPA (1.7 nmol, 1 μg) dissolved in acetone (20 μL) was applied to the right ear only of ICR mice by means of a micropipette. A volume of 10 μL was delivered to both the inner and outer surface of the ear. The samples or their vehicles, CHCl_3_/MeOH (1:1, 20 μL), as a control, were applied topically about 30 min before TPA treatment. For ear thickness determinations, a pocket thickness gauge with a range of 0–9 mm, graduated at 0.01 mm intervals and modified so that the contact surface area was increased to reduce the tension, was applied to the tip of the ear. The ear thickness was measured before treatment (*a*) and 6 h after TPA treatment (*b* = TPA alone; *b*’ = TPA plus sample). The following values were then calculated:

- Edema A as induced by TPA alone (*b*–*a*)
- Edema B as induced by TPA plus sample (*b*’–*a*)
- Inhibitory ratio (IR) (%) = [(Edema A − Edema B)/Edema A] × 100
- Each value was the mean of individual determinations from five mice. The 50% inhibitory dose (ID_50_) values were determined by the method of probit-graphic interpolation for four dose levels.

## 4. Conclusions

In this study, we isolated two new lanostane-type triterpennes, together with 15 known triterpenoids. We evaluated the anti-inflammatory activities of all the tetracyclic triterpenoids. Triterpenoids 1–11 isolated from *E. maculata* exhibited potent anti-inflammatory activities. Many triterpenes with different skeletons, such as oleanae-, ursane-, lupine-, lanostane-, and multiflorane-types, inhibited the tumor promoting activity of TPA. Therefore, these triterpenes might be the candidates for cancer chemopreventive agents.

## Figures and Tables

**Table 1:** Inhibitory effects of triterpenoids on TPA-induced inflammation in mice.

Compounds	ID_50_^a^ (nM/ear)	Range
4-methyl-3,7-dihydroxy-7(8→9) *abeo*-lanost-24(28)-en-8-one (**1**)	803	624–1035
24-hydroperoxylanost-7,25-dien-3β-ol (**2**)	356.3	280.8–452.3
3-hydroxycycloart-25-ene-24-hydroperoxide (**3**)	301.7	212.0–429.3
3β-hydroxy-26-nor-9,19-cyclolanost-23-en-25-one (**4**)	558	462–836
cycloart-23-en-3β,25-diol (**5**)	355.7	276.2–458.1
(23*E*)-3β,25-dihydroxytirucalla-7,23-diene (**6**)	855	644–1134
(23*Z*)-3β,25-dihydroxy-tirucalla-7,23-diene (**7**)	1087	809–1460
Obtusifoliol (**8**)	87.1	36.8–206.4
cycloeucalenol (**9**)	463.9	345.4–623.0
4α,l4α-dimethyl-5α-ergosta-7,9(11),24(28)-trien-3β-ol (**10**)	363.1	273.7–481.6
gramisterol (**11**)	204	86.1–484
Indomethacin ^b^	838.0	730–945

^a^ ID_50_: The 50% inhibitory dose. ^b^ Reference compound.

**Table 2:** ^1^H and ^13^C-NMR Data of compounds **1** and **2** (600 MHz and 150 MHz in CDCl_3_).

Pos.	1		2	
	*δ*_C_, Type	*δ*_H_, m (*J* in Hz)	*δ*_C_, Type	*δ*_H_, m (*J* in Hz)
**1**	30.1, CH_2_	1.59 (m)	37.2, CH_2_	1.13 (m) 1.69 (m)
**2**	30.8, CH_2_	1.70 (m) 1.83 (m)	27.6, CH_2_	1.65 (m)
**3**	77.0, CH	3.05 (td, *J =* 10.0, 4.8 Hz)	79.2, CH	3.24 (dd, *J* = 11.7, 4.6 Hz)
**4**	38.1, CH	1.66 (m)	38.9, C	
**5**	48.2, CH	1.21 (m)	50.6, CH	1.32 (m)
**6**	37.8, CH_2_	1.37 (m) 2.39 (m)	23.9, CH_2_	1.94 (m) 2.15 (m)
**7**	80.5, CH	4.35 (m)	117.8, CH	5.26 (dd, *J* = 7.6, 3.3 Hz)
**8**	215.1, C		145.7, C	
**9**	64.0, C		48.9, CH	2.21 (m)
**10**	48.1, C		34.9, C	
**11**	28.7, CH_2_	1.41 (m)	18.1, CH_2_	0.99 (m) 1.52 (m)
**12**	30.1, CH_2_	1.61 (m) 1.78 (m)	33.7, CH_2_	1.61 (m) 1.80 (m)
**13**	47.6, C		43.5, C	
**14**	61.0, C		51.1, C	
**15**	29.5, CH_2_	1.23 (m) 2.0 (m)	33.9, CH_2_	1.46 (m)
**16**	27.0, CH_2_	1.32 (m) 1.94 (m)	29.9, CH_2_	1.26 (m)
**17**	50.1, CH	1.65 (m)	52.7, CH	1.48 (m)
**18**	16.5, CH_3_	0.66 (s)	21.9, CH_3_	0.81 (s)
**19**	18.4, CH_3_	1.48, s	13.1, CH_3_	0.75 (s)
**20**	35.6, CH	1.43 (m)	36.0, CH	1.37, m
**21**	18.8, CH_3_	0.93 (d, *J* = 6.5 Hz)	18.3, CH_3_	0.87 (d, *J* = 7.4 Hz)
**22**	34.7, CH_2_	1.16 (m) 1.59 (m)	31.8, CH_2_	1.27 (m) 1.42 (m)
**23**	31.2, CH_2_	1.88 (m) 2.13 (m)	28.1, CH_2_	1.67 (m) 1.95 (m)
**24**	156.5, C		90.1, CH	4.21 (t, *J* = 8.0 Hz)
**25**	33.8, CH	2.23 (m)	143.8, C	
**26**	21.9, CH_3_	1.03 (d, *J* = 6.8 Hz)	114.2, CH_2_	5.02 (m)
**27**	22.0, CH_3_	1.02 (d, *J* = 6.7 Hz)	17.1, CH_3_	1.73 (s)
**28**	106.2, CH_2_	4.66 (d, *J* = 1.2 Hz) 4.72 (d, *J* = 0.9 Hz)	27.2, CH_3_	0.98 (s)
**29**	16.2, CH_3_	0.95 (d, *J* = 6.2 Hz)	14.7, CH_3_	0.86 (s)
**30**	19.6 CH_3_	1.21 (s)	27.7, CH_3_	0.98 (s)

## Supplementary Materials

HREIMS, 1D (^1^H-NMR, ^13^C-NMR, and DEPT spectra), and 2D (HMQC, ^1^H-^1^H COSY, HMBC, and NOESY spectra) NMR spectra of new compounds **1** and **2** were available online.

## References

[1] Asgarpour R., Ghorbani R., Khajeh-Hosseini M., Mohammadvand E., Chauhan B.S. (2016). Germination of spotted Spurge (*Chamaesyce maculata*) Seeds in Response to Different Environmental Factors. Weed Sci..

[2] Chinese Herbalism Editorial Board, State Administration of Traditional Chinese Medicine of the People’s Republic of China (1999). Chinese Materia Medica.

[3] Kwon S.U., Cha J.Y., Lee H.Y., Xin M., Ji S.J., Kim D.K., Park D.S., Pyo M.K., Lee Y.M. (2015). Chloroform fraction of *Euphorbia maculata* has antiplatelet activity via suppressing thromboxane B-2 formation. Mol. Med. Rep..

[4] Agata I., Hatano T., Nakaya Y., Sugaya T., Nishibe S., Yoshida T., Okuda T. (1991). Tannins and related polyphenols of Euphorbiaceous plants. 8. Eumaculin A and Eusupinin A, and accompanying polyphenols from *Euphorbia maculata* L. and *E. supina* Rafin. Chem. Pharm. Bull..

[5] Matsunaga S., Tanaka R., Akagi M. (1988). Triterpenoids from *Euphorbia maculata*. Phytochemistry.

[6] Amakura Y., Kawada K., Hatano T., Agata I., Sugaya T., Nishibe S., Okuda T., Yoshida T. (1997). Four new hydrolysable tannins and an acylated flavonol glycoside from *Euphorbia maculata*. Can. J. Chem..

[7] Luyen B.T.T., Tai B.H., Thao N.P., Lee S.H., Jang H.D., Lee Y.M., Kim Y.H. (2014). Evaluation of the anti-osteoporosis and antioxidant activities of phenolic compounds from *Euphorbia maculata*. J. Korean Soc. Appl. Biol..

[8] Yasukawa K. (2013). Horizons in Cancer Research.

[9] Passos G.F., Medeiros R., Marcon R., Nascimento A.F.Z., Calixto J.B., Pianowski L.F. (2013). The role of PKC/ERK1/2 signaling in the anti-inflammatory effect of tetracyclic triterpene euphol on TPA-induced skin inflammation in mice. Eur. J. Pharm..

[10] Fernandez-Arche A., Saenz M.T., Arroyo M., Puerta R., Garcia M.D. (2010). Topical anti-inflammatory effect of tirucallol, a triterpene isolated from *Euphorbia lactea* latex. Phytomedicine.

[11] Ding H.Y., Wu P.S., Wu M.J. (2016). *Cleome rutidosperma* and *Euphorbia thymifolia* suppress inflammatory response via upregulation of phase II enzymes and modulation of NF-κB and JNK activation in LPS-stimulated BV2 microglia. Int. J. Mol. Sci..

[12] Yasukawa K., Akihisa T., Yoshida Z., Takido M. (2000). Inhibitory Effect of Euphol, a Triterpene Alcohol from the Roots of *Euphorbia kansui*, on Tumour Promotion by 12-*O*-Tetradecanoylphorbol-13-acetate in Two-stage Carcinogenesis in Mouse Skin. J. Pharm. Pharmacol..

[13] Kakegawa T., Miyazaki A., Yasukawa K. (2016). Anti-inflammatory effects of alpinone 3-acetate from *Alpinia japonica* seeds. J. Nat. Med..

[14] Yasukawa K., Okada S., Nobushi Y. (2014). Inhibitory effects of Gymnema (*Gymnema sylvestre*) leaves on tumour promotion in two-stage mouse skin carcinogenesis. Evid.-Based Complement. Alternat. Med..

[15] Nakajima J., Nakae D., Yasukawa K. (2013). Structure-dependent inhibitory effects of synthetic cannabinoids against 12-*O*-tetradecanoylphorbol-13-acetate-induced inflammation and skin tumour promotion in mice. J. Pharm. Pharmacol..

[16] Sun Y., Yasukawa K. (2008). New anti-inflammatory ergostane-type ecdysteroids from the sclerotium of *Polyporus Umbellatus*. Bioorg. Med. Chem. Lett..

[17] Oksüz S., Gil R.R., Chai H., Pezzuto J.M., Cordell G.A., Ulubelen A. (1994). Biologically Active Compounds from the Euphorbiaceae; 2. Two Triterpenoids of *Euphorbia cyparissias*. Planta Med..

[18] Parveen M., Khan N.U., Achari B., Dutta P.K. (1991). A Triterpene from *Garcinia mangostana*. Phytochemistry.

[19] Starratt A.N. (1966). Triterpenoid Constituents of *Euphorbia cyparisszas*. Phytochemistry.

[20] Hong Z.L., Xiong J., Wu S.B., Zhu J.J., Zhao Y., Xia G., Hu J.F. (2013). Tetracyclic triterpenoids and terpenylated coumarins from the bark of *Ailanthus altissima* (“Tree of Heaven”). Phytochemistry.

[21] Luo X.D., Wu S.H., Ma Y.B., Wu D.G. (2000). Tirucallane triterpenoids from *Dysoxylum hainanense*. Phytochemistry.

[22] Kemp R.J., Mercer E.I. (1968). The Sterol Esters of Maize Seedlings. Biochem. J..

[23] Brown K.S., Kupchan S.M. (1962). The Structure of Cyclobuxine. J. Am. Chem. Soc..

[24] Akihisa T., Kokke W.C.M.C., Yokota T., Tamura T., Matrumoto T. (1990). 4α, 14α-Dimethyl-5α-ergosta-7, 9(11), 24(28)-trien-3β-ol from *Phaseolus vulgaris* and *Gynostemma pentaphyllum*. Phytochemistry.

[25] Ruiz-Aracama A., Goicoechea E., Guillén M.D. (2017). Direct study of minor extra-virgin olive oil components without any sample modification. ^1^H-NMR multisupression experiment: A powerful tool. Food Chem..

[26] Bohlmann F., Zdero C., King R., Robinson H. (1984). A Hydroxygermacrene and Other Constituents from *Pseudobrickellia brasiliensis*. Phytochemistry.

[27] Yagishita K., Nishimura M. (1961). The Chemical Structure of Neoilexonol. Agric. Biol. Chem..

[28] Yan H.Y., Wang K.W. (2017). Triterpenoids from *Microtropis fokienensis*. Chem. Nat. Compd..

[29] Chiu H.L., Wu J.H., Tung Y.T., Lee T.H., Chien S.C., Kuo Y.H. (2008). Triterpenoids and Aromatics from *Derris laxiflora*. J. Nat. Prod..

[30] Prashant A., Krapadanam G.L.D. (1993). Dehydro-6-hydroxyroxyrotenoid and Lupenone from *Tephrosia villosa*. Phytochemistry.

[31] Hiroyuki A., Yoko A. (1983). Fern constituents: Pentacyclic triterpenoids isolated from *Polypodium niponicum* and *P. formosalem*. Phytochemistry.

[32] Daoubi M., Marquez N., Mazoir N., Benharref A., Hernandez-Galan R., Munoz E., Collado I.G. (2007). Isolation of new phenylacetylingol derivatives that reactivate HIV-1 latency and a novel spirotriterpenoid from *Euphorbia officinarum* latex. Bioorg. Med. Chem..

[33] Xia Q., Zhang H., Sun X., Zhao H., Wu L., Zhu D., Yang G., Shao Y., Zhang X., Mao X. (2014). A comprehensive review of the structure elucidation and biological activity of triterpenoids from *Ganoderma* spp.. Molecules.

[34] Yasukawa K., Yu S.Y., Yamanouchi S., Takido M., Akihisa T., Tamura T. (1995). Some lupine-type triterpenes inhibit tumor promotion by 12-*O*-tetradecanoylphorbol-13-acetate in two-stage carcinogenesis in mouse skin. Phytomedicine.

