# Supplementary material for: Triterpenoids from Euphorbia maculata and Their Anti-Inflammatory Effects
Source: Molecules. 2018 Aug 22;23(9):2112. doi: 10.3390/molecules23092112 (PMC6225269; doi:10.3390/molecules23092112)
Supplement: Supplementary file 1 [file molecules-23-02112-s001.pdf]

# Supplementary Information

## Triterpenoids from *Euphorbia maculata* and their Anti-Inflammatory Effects

**Yi Sun[1,2]*, Liangliang Gao[3], Mengyue Tang[1,4], Baomin Feng[5], Yuehu Pei[4] and Ken Yasukawa[2,]***

[1] Institute of Chinese Materia Medica, China Academy of Chinese Medical Sciences, Beijing 100700, China; m18656251050@163.com (Y.T.)

[2] School of Pharmacy, Nihon University, 7-7-1, Narashinodai, Funabashi, Chiba 274-8555, Japan

[3] College of Food and Medicine, Anhui Science and Technology University, Fengyang 233100, China; Gll8179@163.com

[4] Pharmacy College, Harbing Medical University, Harbin 150081, China; fengbaomin@dlu.edu.com

[5] School of Life and Sciences and Biotechnology, Dalian University, Dalian 116622, China

* Correspondence: ysun@icmm.ac.cn (Y.S.); peiyueh@vip.163.com (Y.P.); yasukawa.ken@nihon-u.ne.jp (K.Y.); Tel.:+86-10-64032656 (Y.S.)

Figure S1: $^{1}H$ NMR spectrum (600 MHz, $CDCl_3$) of compound **1**

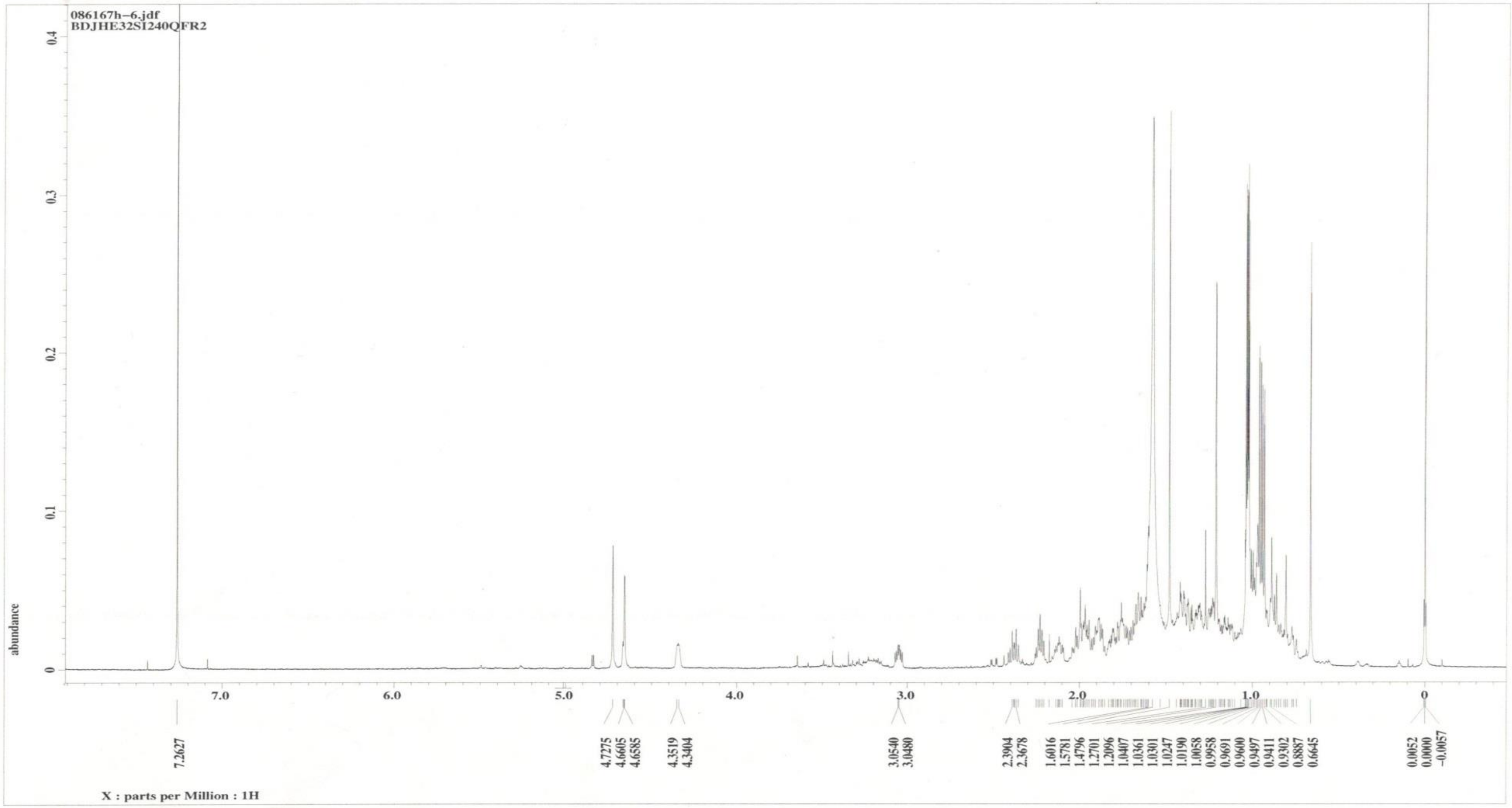

Figure S2: $^{13}$C NMR spectrum (150 MHz, $CDCl_3$) of compound **1**

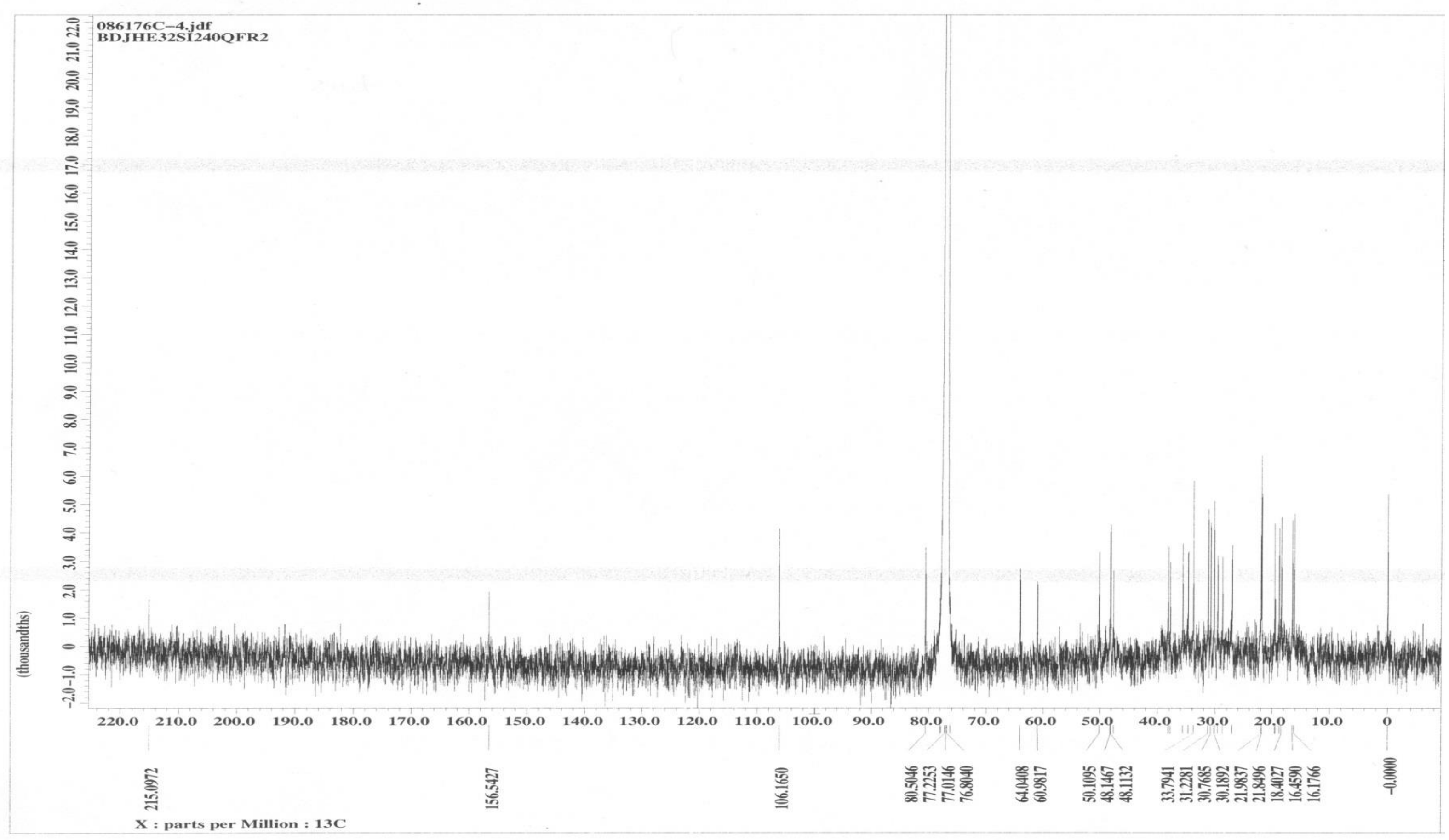

Figure S3: HMQC spectrum (600 MHz, $CDCl_3$) of compound **1**

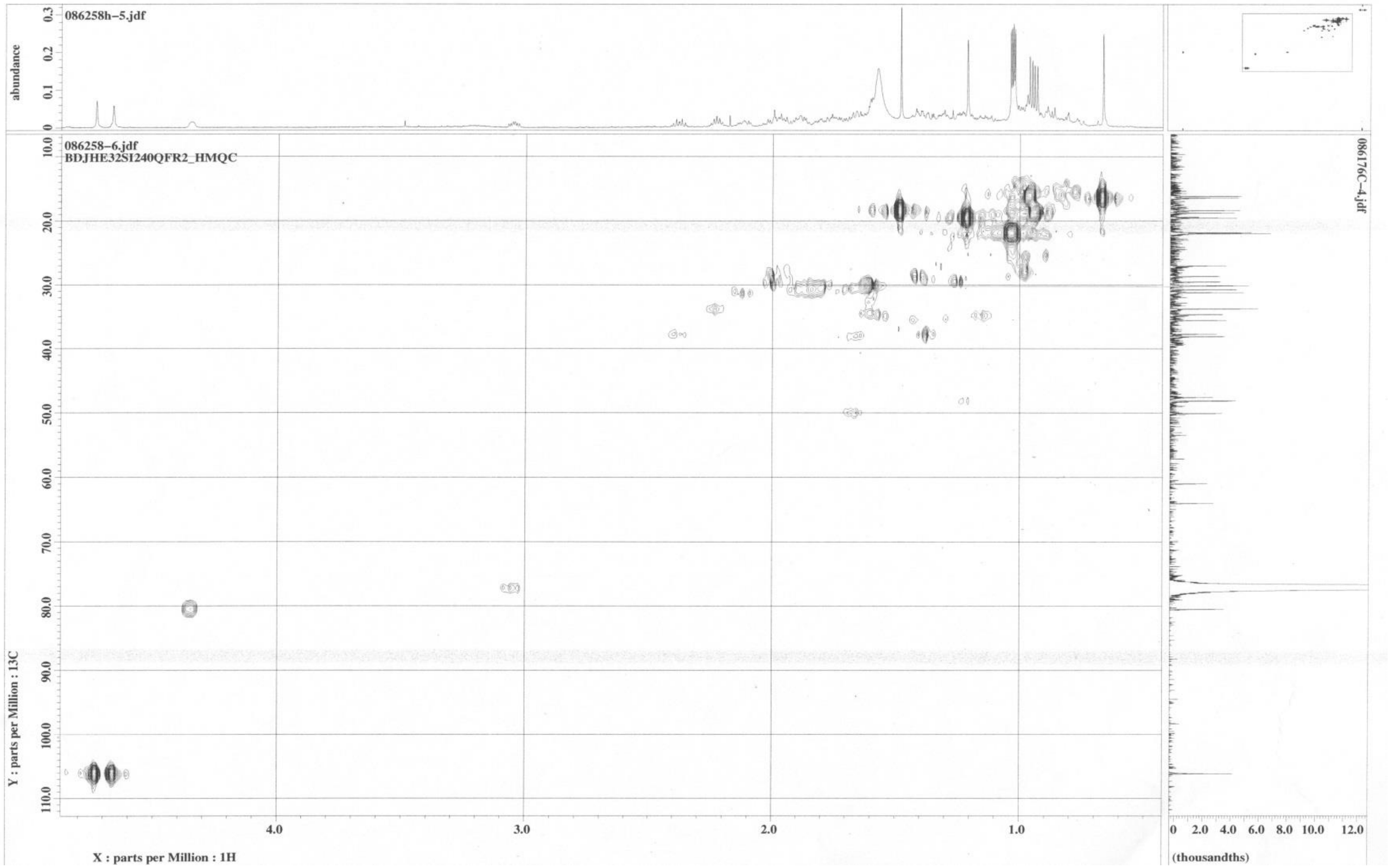

Figure S4: HMBC spectrum (600 MHz, $CDCl_3$) of compound **1**

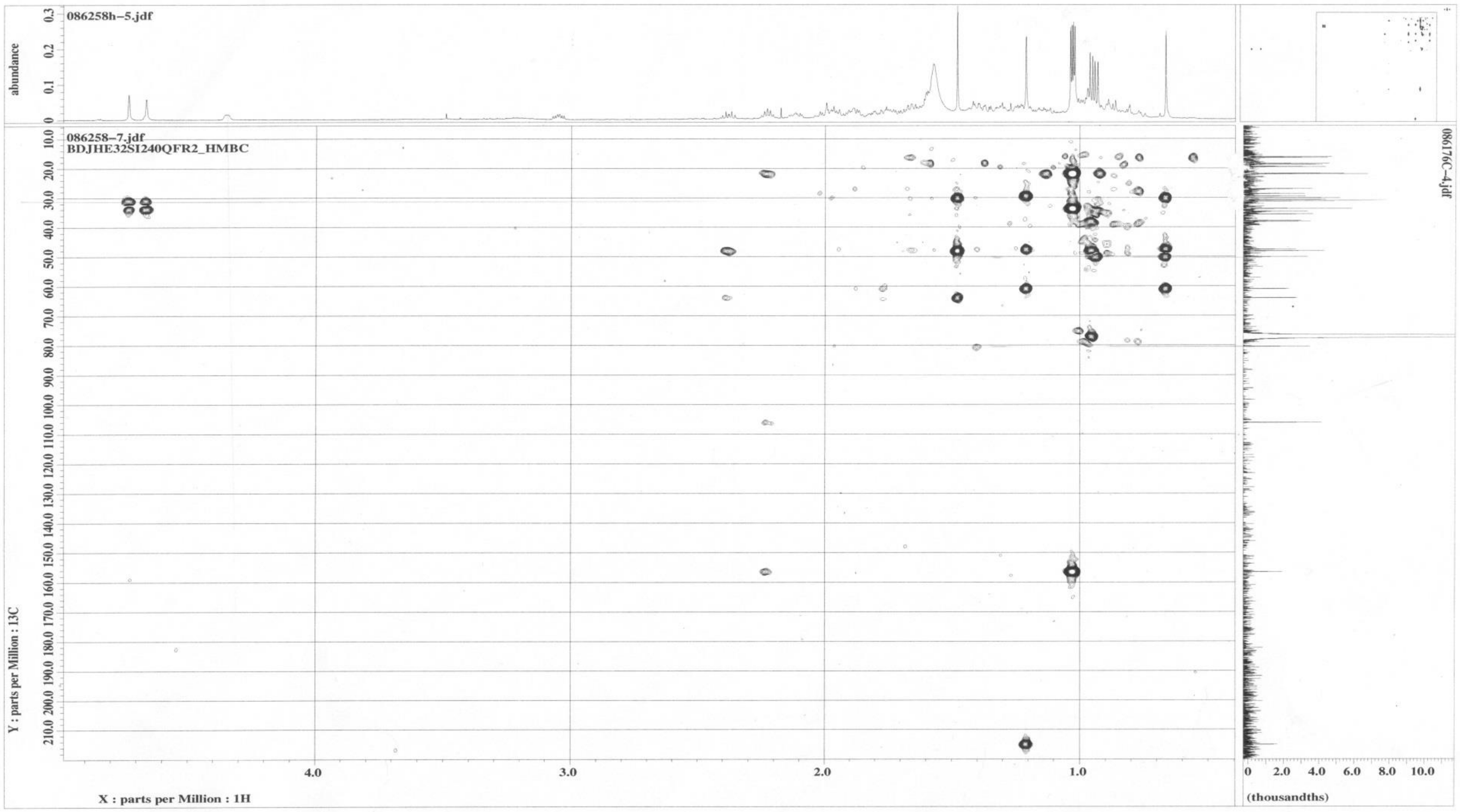

Figure S5: $^{1}H$-$^{1}H$ COSY spectrum (600 MHz, $CDCl_3$) of compound **1**

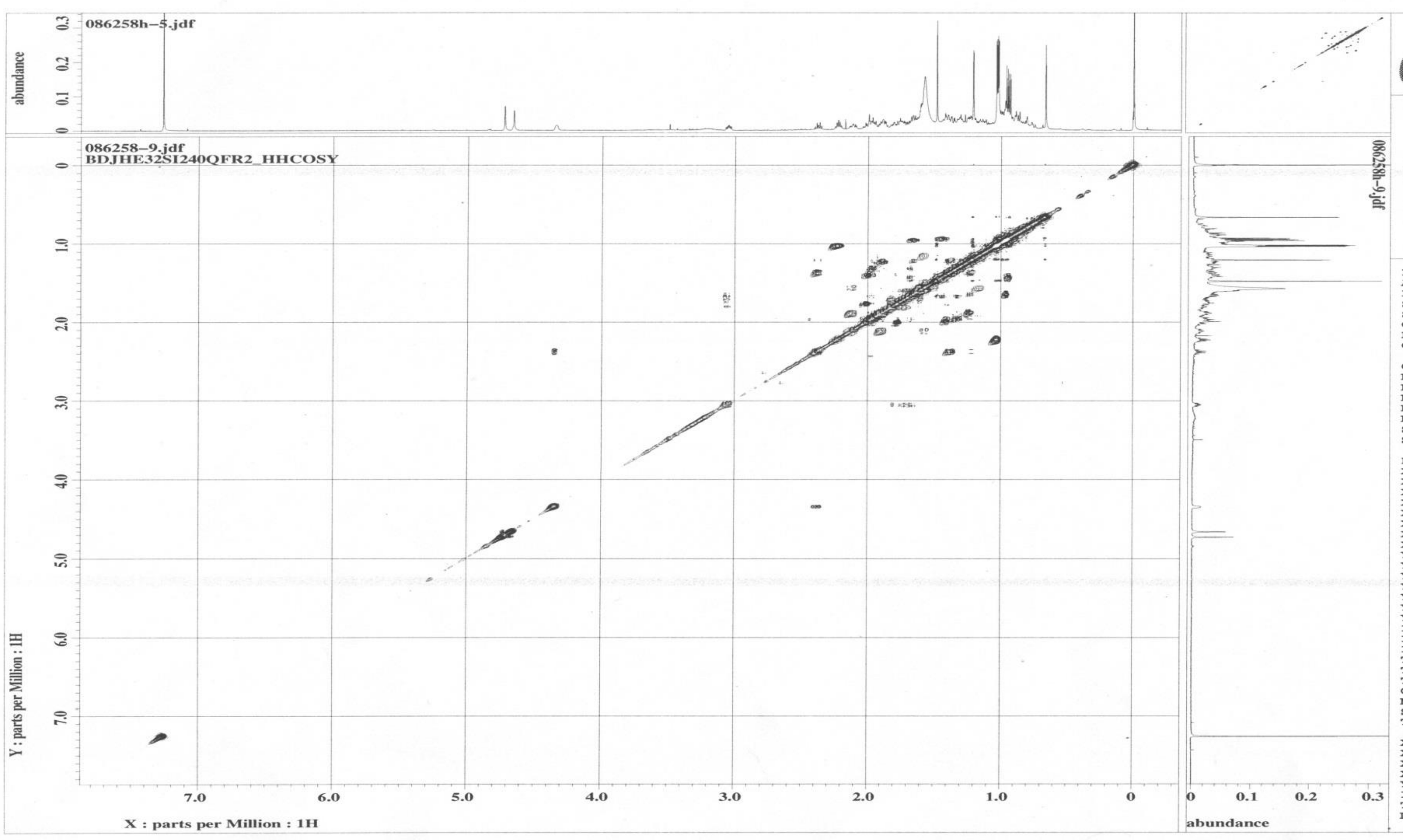

Figure S6: NOESY spectrum (600 MHz, $CDCl_3$) of compound **1**

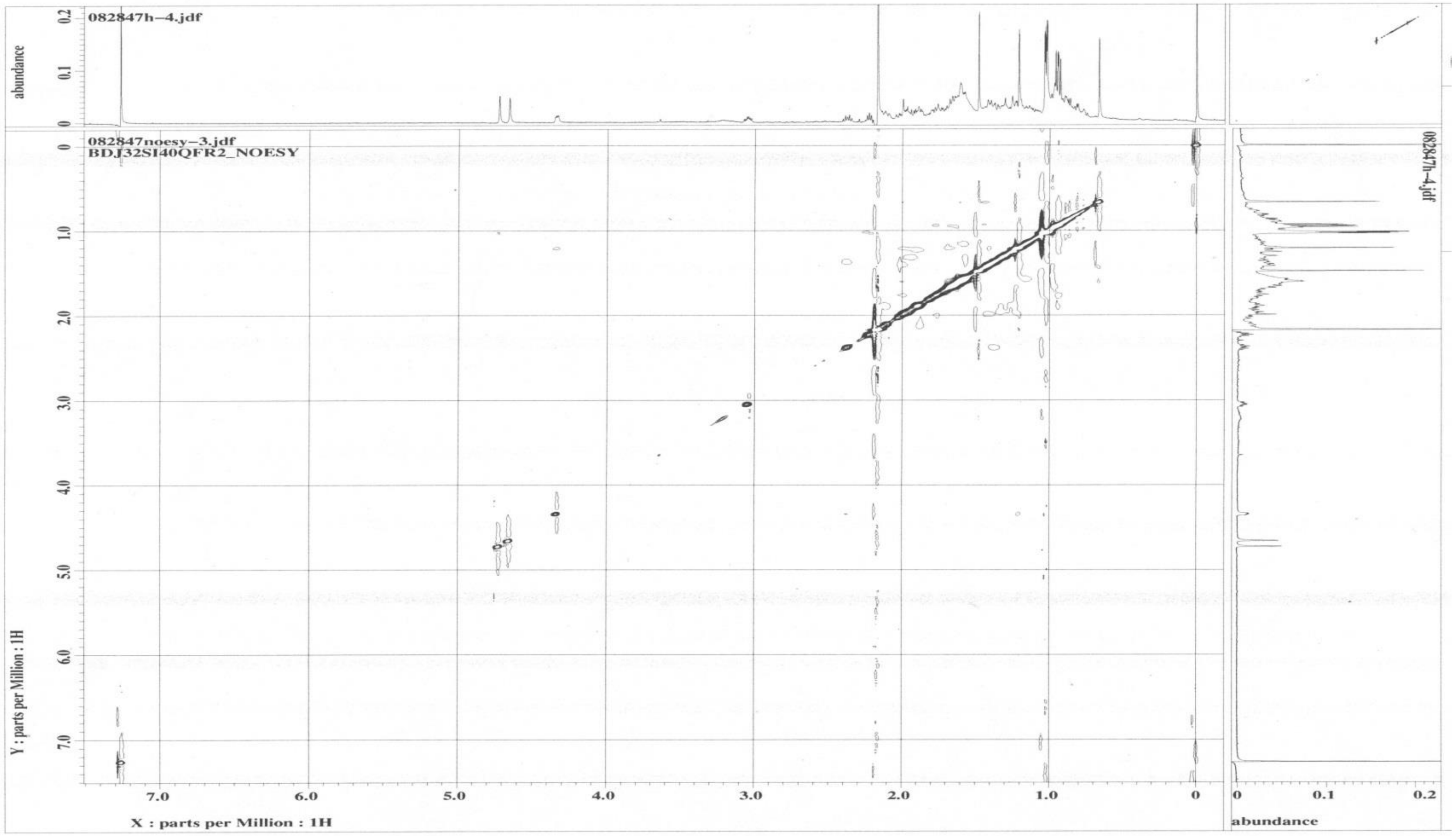

Figure S7: $^{1}H$ spectrum (600 MHz, $CDCl_3$) of compound **2**

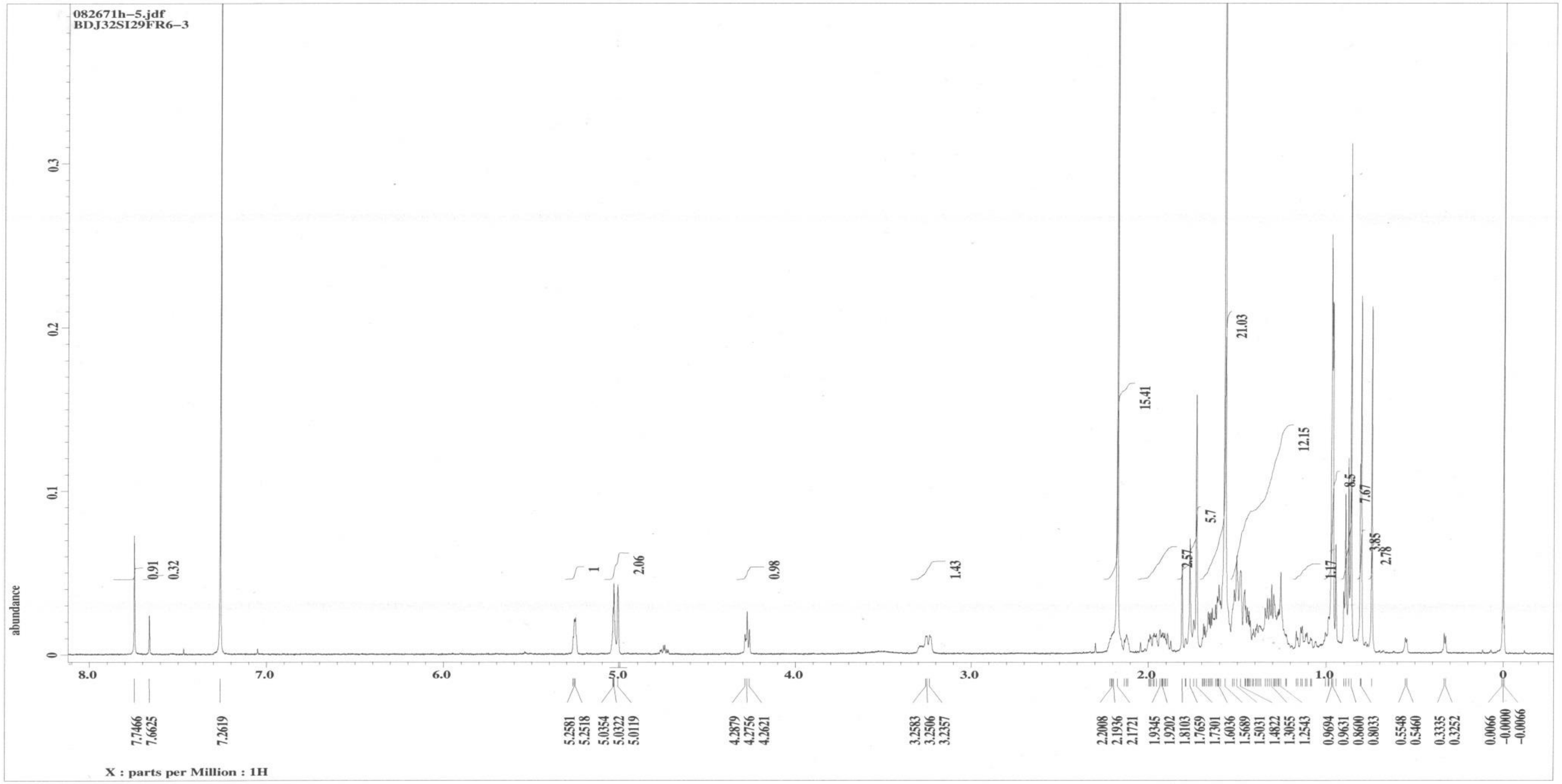

Figure S8: $^{13}$C spectrum (150 MHz, $CDCl_3$) of compound **2**

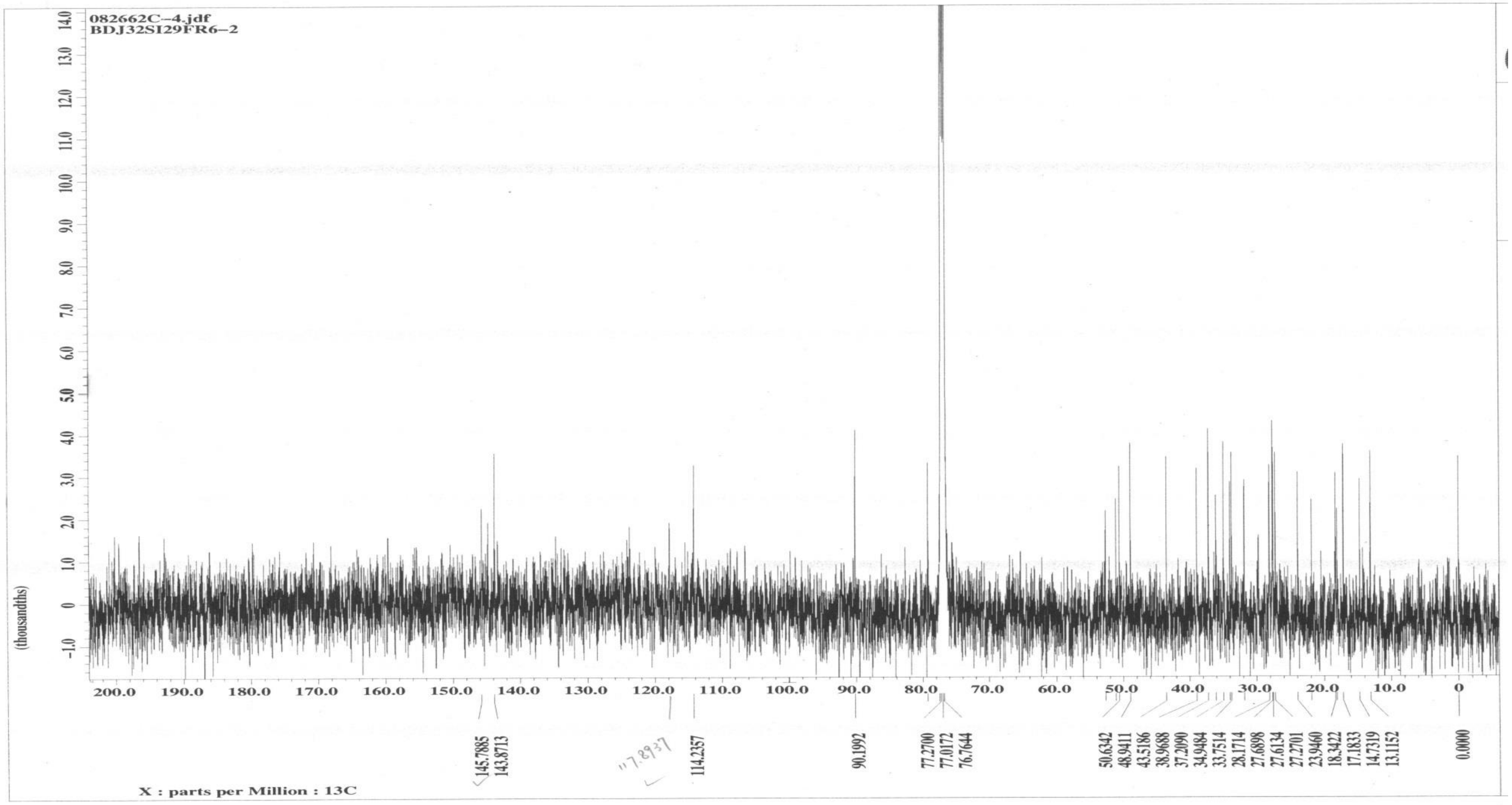

Figure S9: HMQC spectrum (600 MHz, $CDCl_3$) of compound **2**

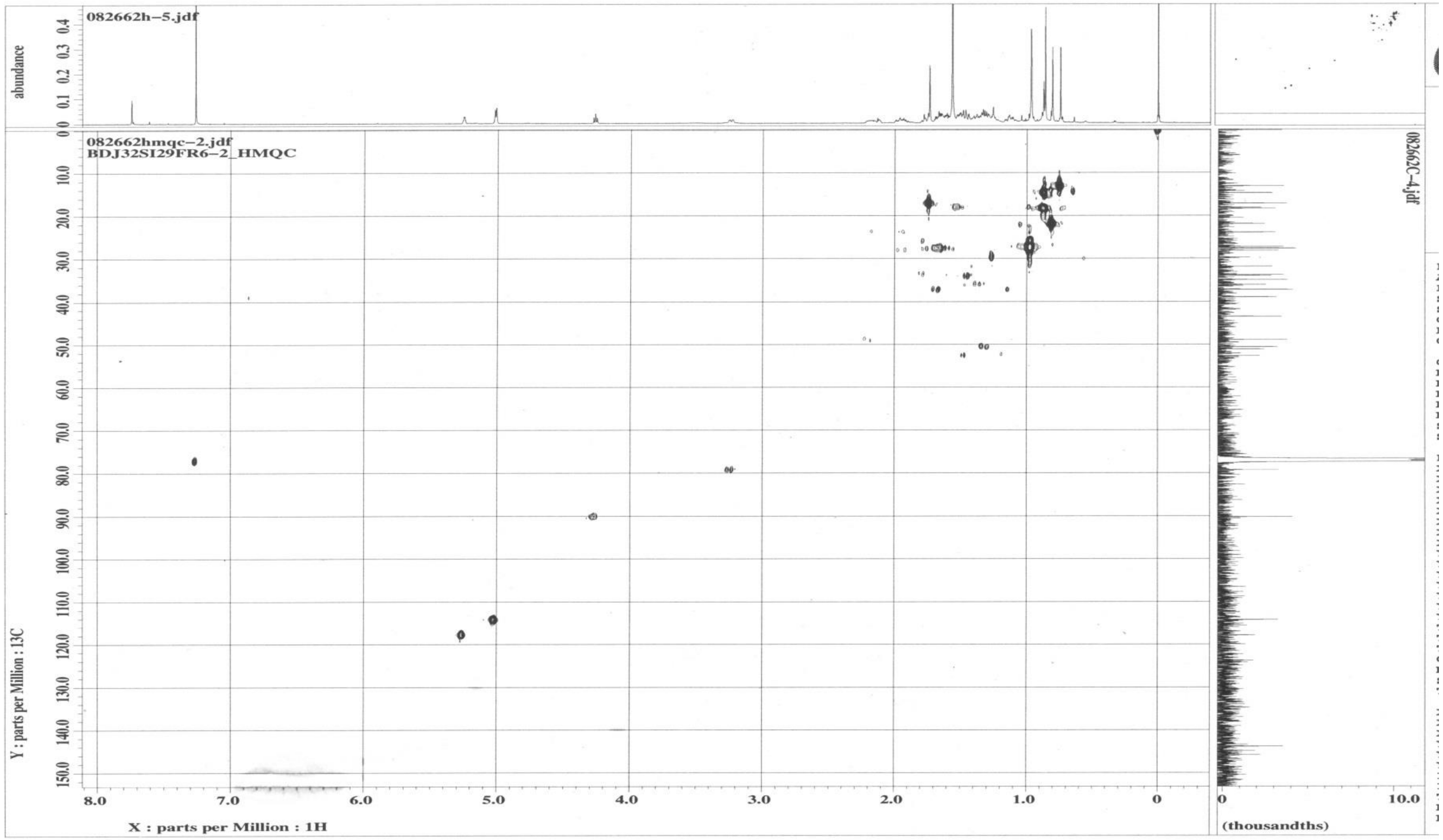

Figure S10: HMBC spectrum (600 MHz, $CDCl_3$) of compound **2**

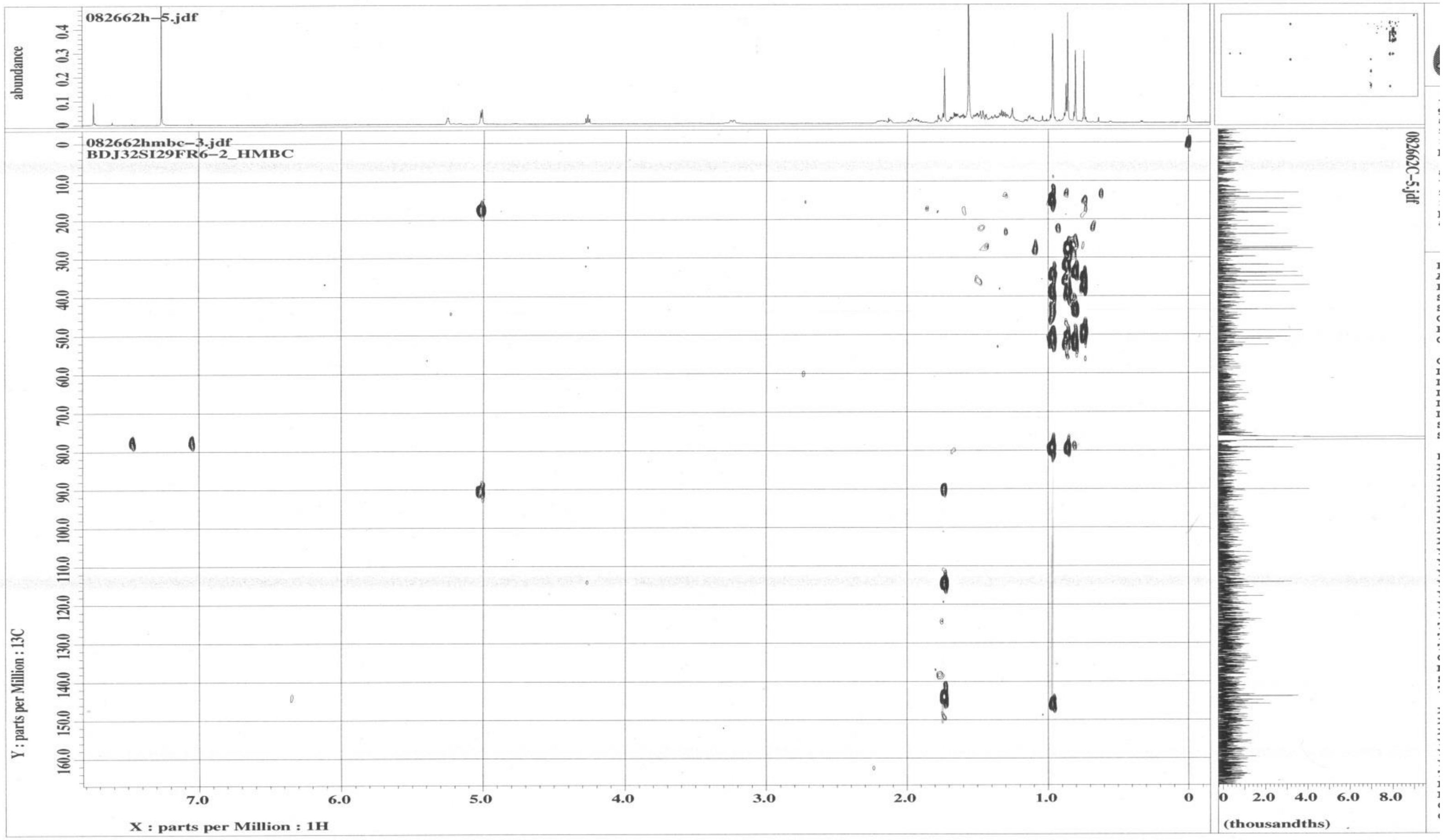

Figure S11: $^{1}$H-$^{1}$H COSY spectrum (600 MHz, $CDCl_3$) of compound **2**

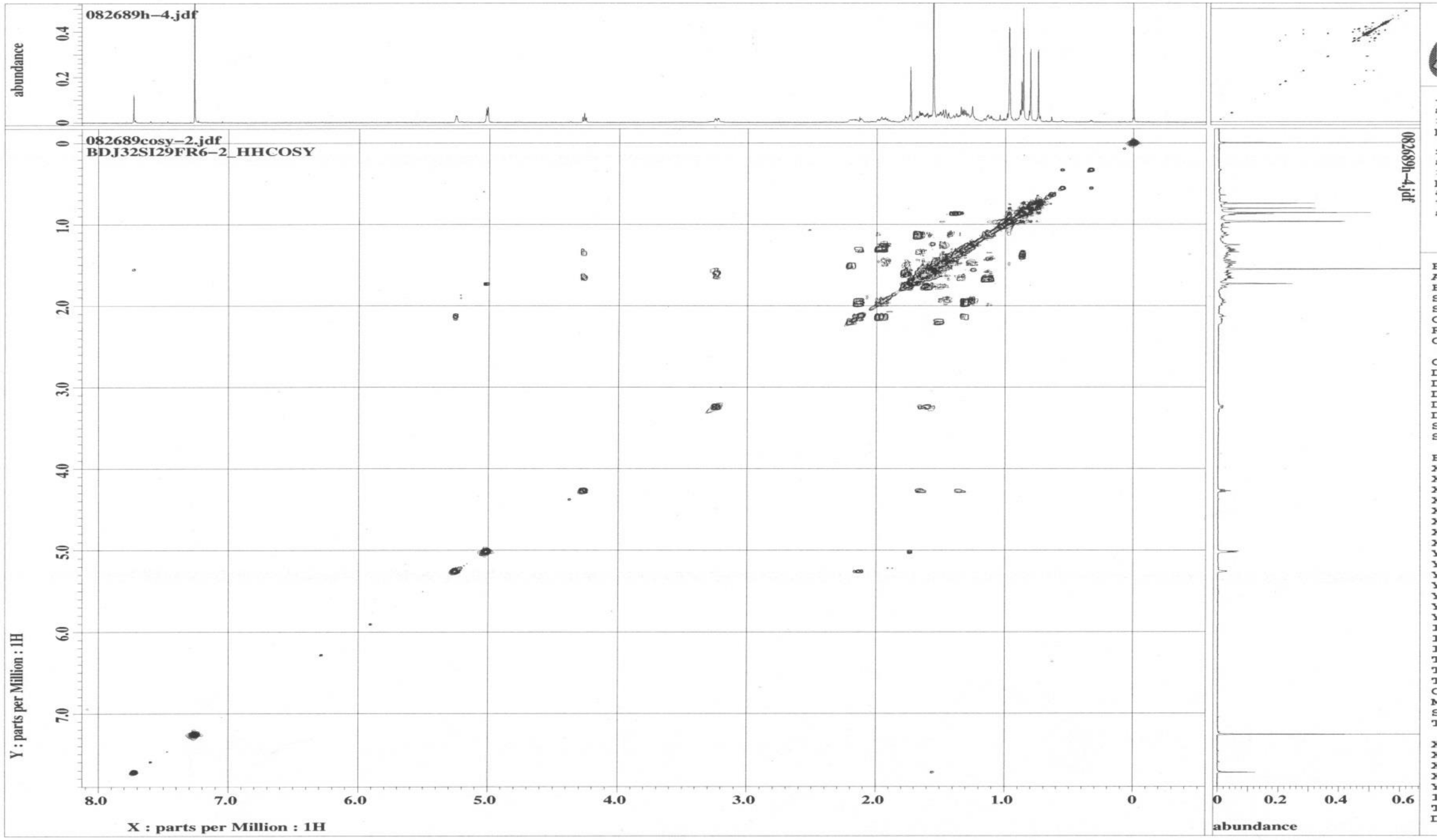

Figure S12: NOESY spectrum (600 MHz, $CDCl_3$) of compound **2**

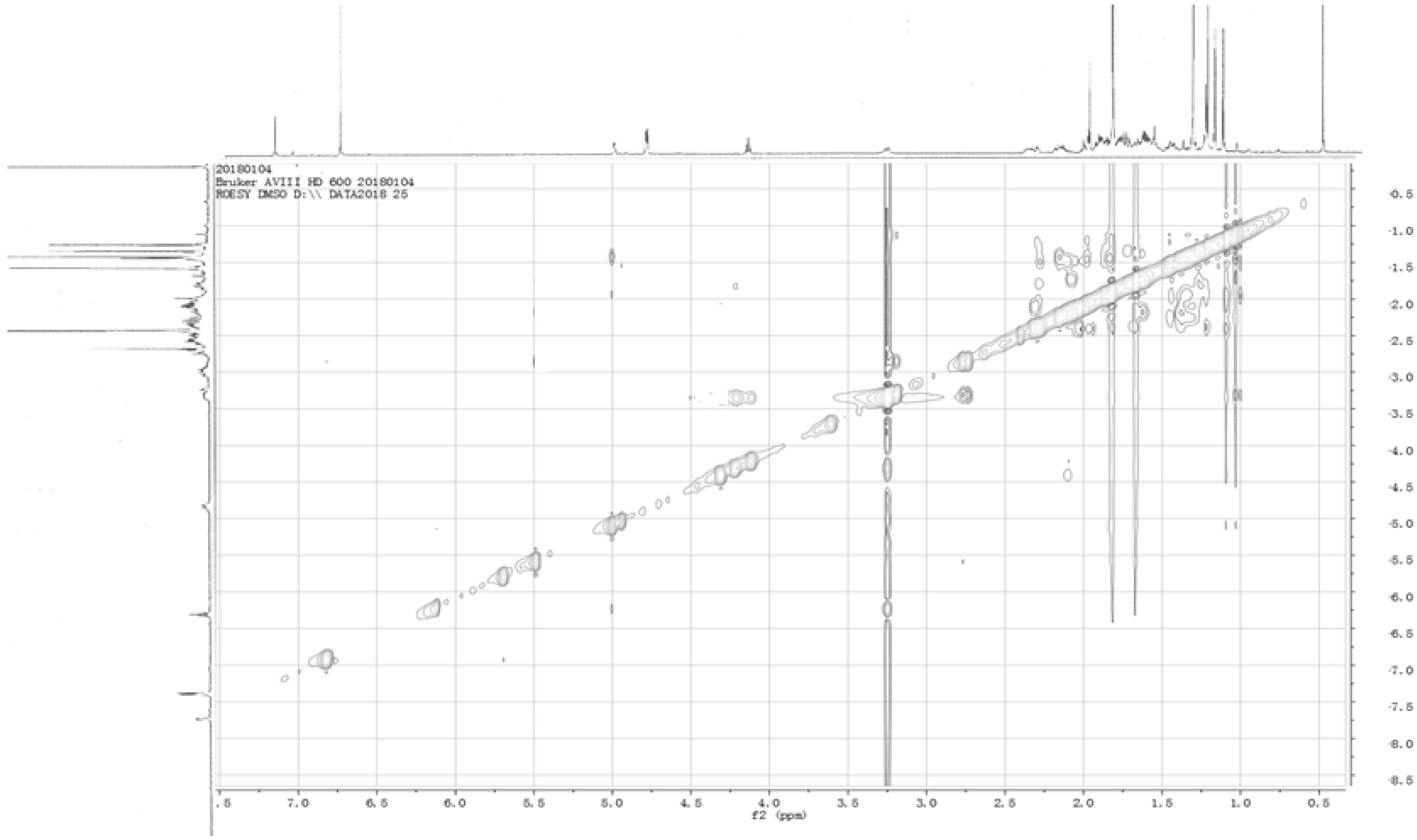

Figure S13: EI MS spectrum of compound **1**

Scan: 36
Base: m/z 458; 1.1%FS TIC: 203733

R.T.: 2:21

#Ions: 163

Selected Isotopes : $H_{0\text{-}55}C_{0\text{-}35}{}^{13}C_{0\text{-}2}O_{0\text{-}5}$ Error Limit : 10 mmu Unsaturation Limits : -.5 - 30

| Measured Mass | % Base | Formula | Calculated Mass | Error | Unsaturation |
|---|---|---|---|---|---|
| 458.37593 | 100.0% | $C_{32}H_{48}{}^{13}C_2$ | 458.38230 | 6.4 | 11.0 |
| | | ✓ $C_{30}H_{50}O_3$ | 458.37598 | 0.1 | 6.0 |
| | | $C_{29}H_{49}{}^{13}C\,O_3$ | 458.37150 | -4.4 | 6.5 |
| | | $C_{28}H_{48}{}^{13}C_2O_3$ | 458.36703 | -8.9 | 7.0 |
| 459.38013 | 27.9% | $C_{32}H_{49}{}^{13}C_2$ | 459.39013 | 10.0 | 10.5 |
| | | $C_{30}H_{51}O_3$ | 459.38380 | 3.7 | 5.5 |
| | | $C_{29}H_{50}{}^{13}C\,O_3$ | 459.37933 | -0.8 | 6.0 |
| | | $C_{28}H_{49}{}^{13}C_2O_3$ | 459.37485 | -5.3 | 6.5 |
| 460.37939 | 8.8% | $C_{33}H_{48}O$ | 460.37051 | -8.9 | 10.0 |
| | | $C_{29}H_{51}{}^{13}C\,O_3$ | 460.38716 | 7.8 | 5.5 |
| | | $C_{28}H_{50}{}^{13}C_2O_3$ | 460.38268 | 3.3 | 6.0 |

Figure S14: EI MS spectrum of compound **2**

Scan: 21
Base: m/z 458; .9%FS TIC: 351334

R.T.: 1:21.1

#Ions: 250

Selected Isotopes : $H_{0\text{-}60}C_{0\text{-}35}{}^{13}C_{0\text{-}2}O_{0\text{-}5}$

Error Limit : 10 mmu

Unsaturation Limits : -.5 - 30

| Measured Mass | % Base | Formula | Calculated Mass | Error | Unsaturation |
|---|---|---|---|---|---|
| 458.37554 | 100.0% | $C_{32}H_{48}{}^{13}C_2$ | 458.38230 | 6.8 | 11.0 |
| | | $C_{30}H_{50}O_3$ | 458.37598 | 0.4 | 6.0 |
| | | $C_{29}H_{49}{}^{13}C\,O_3$ | 458.37150 | -4.0 | 6.5 |
| | | $C_{28}H_{48}{}^{13}C_2O_3$ | 458.36703 | -8.5 | 7.0 |
| 459.37072 | 38.4% | $C_{33}H_{47}O$ | 459.36268 | -8.0 | 10.5 |
| | | $C_{29}H_{50}{}^{13}C\,O_3$ | 459.37933 | 8.6 | 6.0 |
| | | $C_{28}H_{49}{}^{13}C_2O_3$ | 459.37485 | 4.1 | 6.5 |
| 460.37846 | 8.3% | $C_{33}H_{48}O$ | 460.37051 | -8.0 | 10.0 |
| | | $C_{29}H_{51}{}^{13}C\,O_3$ | 460.38716 | 8.7 | 5.5 |
| | | $C_{28}H_{50}{}^{13}C_2O_3$ | 460.38268 | 4.2 | 6.0 |
